# Peer and land-based approaches for fostering empowering and healthy relationships with Indigenous and northern young people in the Northwest Territories

**DOI:** 10.1371/journal.pone.0298166

**Published:** 2024-04-05

**Authors:** Lesley Gittings, Kalonde Malama, Carmen Logie, Candice Lys, Shira B. Taylor, Clara McNamee, Kayley Inuksuk Mackay, Zerihun Admassu

**Affiliations:** 1 Faculty of Health Sciences, School of Health Studies, Western University, London, Ontario, Canada; 2 University of Cape Town Centre for Social Science Research, Cape Town, Western Cape, South Africa; 3 Children’s Health Research Institute, London, Ontario, Canada; 4 Factor-Inwentash Faculty of Social Work, University of Toronto, Toronto, Ontario, Canada; 5 Women’s College Research Institute, Women’s College Hospital, Toronto, Ontario, Canada; 6 Centre for Gender & Sexual Health Equity, Vancouver, British Columbia, Canada; 7 Fostering Open eXpression Among Youth (FOXY), Yellowknife, NT, Canada; 8 Aurora College, Yellowknife, NT, Canada; 9 SExT: Sex Education by Theatre, Toronto, Canada; 10 Faculty of Environment and Urban Change, York University, Toronto, ON, Canada; Caleb University, NIGERIA

## Abstract

Indigenous and Northern women in Canada experience high rates of intimate partner violence (IPV), and this is particularly true in the Northwest Territories (NWT). Adolescents are also at increased risk of IPV, which has far-reaching, lifelong effects. Indigenous youth are particularly vulnerable to IPV due to ongoing effects of intergenerational trauma caused by colonialism, racism and residential school legacies. We explored attitudes towards IPV and the healthy relationship knowledge, skills, and experiences among participants of Fostering Open eXpression among Youth (FOXY) and Strength, Masculinities, and Sexual Health (SMASH) Peer Leader Retreats in the NWT. Multi-method approaches included quantitative surveys youth completed before and immediately following retreats. Quantitative analysis from retreats (2018–2021) included 240 participants aged 12–19 (mean age 14.5) who reported ever having an intimate partner. Most were from the FOXY program (64.2%), Indigenous (69.6%) and heterosexual (66.4%). Qualitative methods included Focus Group Discussions (FGD) (n = 69) conducted with peer leaders and apprentices (n = 311) and youth and adult staff (n = 14 FGDs, n = 165 participants). We thematically analysed FGDs to explore healthy relationship knowledge and skills, alongside paired t-tests to examine pre/post retreat changes in attitudes towards IPV. Qualitative findings suggest that leadership and embodied learning were effective in equipping youth with violence prevention and healthy relationship skills. While young women were committed to sharing knowledge and skills about healthy relationships in their communities, young men resonated with values of respect and appreciated support to identify and express emotions. Participants across programmes demonstrated their belief that healthy intimate relationships have communal, relational and intergenerational benefits. Quantitatively, we found a statistically significant reduction in attitudes accepting of IPV among young women, but no changes were noted among young men. Findings contribute to emergent evidence on strengths-based, culturally-responsive IPV prevention programming. Components of effective IPV prevention programming with young men merit further exploration.

## Introduction

Indigenous girls and women in Canada are at the highest risk of violence victimization of all population groups in the country [1]. Intimate partner violence (IPV)–defined as ‘violence committed by a current or former legally married or common-law spouse, or dating partner’ [2]–is among the most pervasive forms of violence against women [3], and is a major element of violence experienced by Indigenous women. Indigenous women are significantly more likely that non-Indigenous women in Canada to experience all forms of IPV, with 59% of First Nations, 64% of Mátis and 44% of Inuit women reporting experiencing psychological, physical or sexual violence by an intimate partner in their lifetime [4]. This epidemic of IPV is among the deep reaching intergenerational harms caused by colonialism, residential schools, the Indian Act and state enforced erasure of traditions and culture [2,5–8]. It is particularly important to understand IPV in Arctic contexts such as the Northwest Territories (NWT), Canada where girls and women are disproportionately affected.

Prevalence of IPV in NWT are among the highest in Canada, up to ten times higher than the rest of the country [9]. In 2019, the NWT saw 4,083 reports of IPV per 100,000, although such reports are largely a gross underestimate of IPV occurrences [3]. Indigenous peoples comprise a majority of the NWT population and are significantly overrepresented in experiences of IPV of all forms [4,10]. IPV is gendered, and women and girls report IPV in NWT at a rate 2.2 times higher than men and boys [3]. It is urgent to address IPV among young people in the NWT, as adolescence is also a time of heightened risk of IPV—in Canada, young people aged 15–24 are more than twice as likely as the general adult population to have experienced recent IPV [10].

While less data is available on prevalence of IPV among Indigenous and Northern young people in Canada, a recent study among 240 youth in NWT found high prevalence of experiencing (62%) and perpetrating (55%) IPV [11]. Adolescence is an important time in the life course, comprised of major social and developmental transitions with lifelong implications. While risk for IPV victimization and perpetration increases throughout adolescence into emerging adulthood, and then decreases, risk can remain high for those who have survived IPV [12]. Experiencing IPV in adolescence is associated with adult experiences of IPV [13] and depression [13]. IPV has significant and long-lasting impacts on survivors, families and communities [2]. Long-term health impacts of IPV victimization include adverse psychological consequences including depression, suicidality and post-traumatic stress disorder [14], as well as a range of other health issues such as hypertension and chronic pain [15]. The long term impacts of IPV among adolescence underscores the critical need for contextually, gender, culturally appropriate prevention strategies.

Emergent evidence points to the benefit of strengths-based, culturally relevant approaches in health programming with Northern and Indigenous youth. This is needed as a counterpoint to deficits-based research with young people that can frame youth as risk takers, and lacking the skills to care for their health and well-being [16]. Similarly, deficits-based approaches are common in Indigenous health research and practice [17] which marginalise the voices of Indigenous people [18–22]. Despite this, Indigenous youth continue to demonstrate tremendous creativity and resilience. Approaches such as peer-led, art- and land-based youth programming can be empowering [23], and can support coping,[22] resilience, connectedness and self-efficacy for Indigenous and Northern Youth [24]. Such approaches have been documented as transformative, with the potential to catalyze social change and promote social inclusion in diverse global contexts [25]. The importance of culturally-, socially- and historically-responsive violence prevention and healing strategies for youth are being increasingly recognised in research and policy [4]. While strengths-based approaches to healthy relationships hold the potential to advance the health and wellbeing of Northern and Indigenous adolescents, less is known about youth experiences of such programming and their promise in advancing IPV prevention in the NWT.

In response to this critical knowledge gap, this study examines the role of Peer Leader Retreats in addressing attitudes towards IPV and in instilling the knowledge and skills to promote healthy relationships with youth in NWT. The study aims were two-fold. First, we use qualitative analysis from focus groups to explore and document the knowledge, experiences, and perspectives of youth participants in Peer Leader Retreats. Second, we use quantitative analysis to determine pre -post Peer Leader Retreat changes in IPV attitudes by gender. We apply findings to Michau and colleagues’ *prevention of violence against women and girls framework* [26] to conceptualise the inter-related individual, interpersonal and communal aspects of healthy relationship promotion and IPV prevention among youth participants of Peer Leader Retreats in the NWT.

## Materials and methods

Fostering Open eXpression among Youth (FOXY) [24] and Strength, Masculinities, and Sexual Health (SMASH) [27] deliver Peer Leader Retreats in the NWT [24]. As an action research program, they aim to promote healthy relationships, sexual health, and resilience among Northern and Indigenous adolescents using a peer model [24]. FOXY and SMASH nine-day retreats were held annually with adolescents aged 12–19 between 2017 and 2021 in NWT. Retreat participants were recruited using purposive and peer-driven recruitment strategies, including through school, community partners, peer networks, community agencies and over social media in NWT, Yukon and Nunavut between 2017 and 2021. Inclusion criteria were: aged 13–17, living in NWT, Yukon or Nunavut and providing parental consent and youth assent.

FOXY and SMASH Peer Leader Retreats were designed by two lifelong Northerners, one of whom is Indigenous. They were delivered by a group of rotating facilitators, most of whom are from Northern Canada and/or Indigenous. The facilitators at the FOXY Peer Leader Retreats were women and non-binary individuals, while SMASH Peer Leader Retreat facilitators were men, women, and gender diverse people. Retreat leadership also included peer apprentices (volunteer) and peer facilitators (staff), aged 14–21 who had previously attended the retreats as Peer Leaders. Peer Leader Retreat programs included ceremonies, Elder teachings, sharing circles, grief circles, digital storytelling, rattle-making, beading, traditional hand drumming, photography, visual arts, Northern games, sexual and mental health education, journaling, personality mapping and community leadership projects. Design and findings from these programs have been documented elsewhere [23,24,28,29]. FOXY and SMASH are funded by a government and non-governmental research and programmatic funders [30,31].

### Data collection

Quantitative retreat data were collected using a single-group, pre-post test design. A paper-based questionnaire was administered to Peer Leader Retreat participants by an evaluation consultant immediately before, and directly after attending the retreat to assess outcome changes following retreat participation and socio-demographic characteristics (e.g., age, gender, and ethnicity).

Each participant was invited to participate in a focus group discussion (FGD) at the end of the retreat. Focus group discussions (FGDs) were conducted in 2017–2021 with 311 participants, including peer leaders and apprentices (n = 69 groups; ages:12–19) and peer and adult facilitators (n = 14 focus groups; n = 165 facilitator participants). FGD participants were peer leaders, apprentices (returning peer leaders), and facilitators (including youth and adult staff facilitators). FGD questions focused on retreat experiences, relationships skills, leadership, observations of changes in self, healthy relationships and sexual health knowledge. FGDs were recorded with participant consent and transcribed verbatim. Further details of retreat participants demographics can be found in **Table 1**.

**Table 1:** Focus groups by group type and year (2017–2021).

Group type	Year	2017	2018	2019	2020	2021	Total (n = 69)
FOXY Peer Leader		6	6	3	6	6	27
FOXY Peer Apprentice		2	1	1	2	2	8
FOXY Facilitator		2	2	1	2	2	9
SMASH Peer Leader		3	3	3	3	3	15
SMASH Peer Apprentice		1	1	1	1	1	5
SMASH Facilitator		1	1	1	1	1	5

### Qualitative analysis

We took an inductive, data-driven approach and applied thematic analysis [32] to FGD data from 2017–2021 to explore Peer Leader Retreat experiences in relation to healthy relationships and violence prevention programming. After data familiarisation through reviewing transcripts, we generated an initial list of codes related to healthy relationships and violence, and then reviewed, defined and named themes. Analysis was informed by a strengths-based approach that embraces individual and cultural strengths [33].

### Quantitative measures

Attitudes about IPV were assessed before and after the retreat from 2018–2020 using the 10-item Acceptance of Dating Violence scale [34]. Items captured included attitudes towards two types of IPV: physical violence (e.g., hitting), and sexual violence (e.g., forcing sexual intercourse). IPV attitudes were assessed before and after the retreat in 2021 with a different 11-item intimate partner violence attitude scale, the Acceptance of Violence Scale [35]. In both scales, items captured included attitudes towards two types of IPV: physical violence (e.g., hitting), and sexual violence (e.g., forcing sexual intercourse). The overall IPV attitude score was calculated by taking the average of the scale scores, with higher scores indicating greater acceptance of IPV attitudes (2018–2020). Item 10 was omitted from analyses due to its poor psychometric properties, in line with previous research [34]. For the new scale (used in 2021) it was calculated by adding the scores for all 11 items. IPV attitudes were averaged on a scale from 1 to 6 on the 10-item scale and from 11 to 33 on the 11-item scale, with higher scores indicating higher levels of accepting attitudes toward intimate partner violence.

### Statistical analysis

Analyses were conducted using STATA 17. First, we performed descriptive analysis to examine distribution frequencies for categorical variables, and means, standard deviations, and ranges for continuous variables across the total sample. For our outcome, we constructed an additive scale with all IPV attitude items and calculated the average level of approval for each item of the IPV attitudes scale. We tested the internal consistency of the IPV attitudes scale as a whole, and for young men (SMASH) and young women (FOXY) participants separately. Item 10 of the old IPV attitudes scale was excluded from the analysis due to data entry errors. Using the pre- and post-training data, a paired-sample t-test was performed to determine changes in attitudes towards IPV among youth. Our pre-post analysis was gender disaggregated to determine differences between SMASH and FOXY participants.

### Ethics

Ethical approval for this study was obtained from the University of Toronto Research Ethics Board (Protocol #00038346) and the Aurora Research Institute. All participants provided voluntary, informed written consent. Parents/guardians provided reverse consent, in which they would only indicate if they did not wish for their child to participate. Only evaluators who conducted focus groups and administered surveys had access to a participant roster linking individual identifiers to a participant number. Unique participant numbers were used during analysis to link survey participants. In presenting qualitative findings below, we specify whether participants were FOXY or SMASH attendees: FOXY participants included young women, and femme-identified young people, and SMASH participants included young men and masc-identified young people.

## Qualitative results

**Fig 1:**
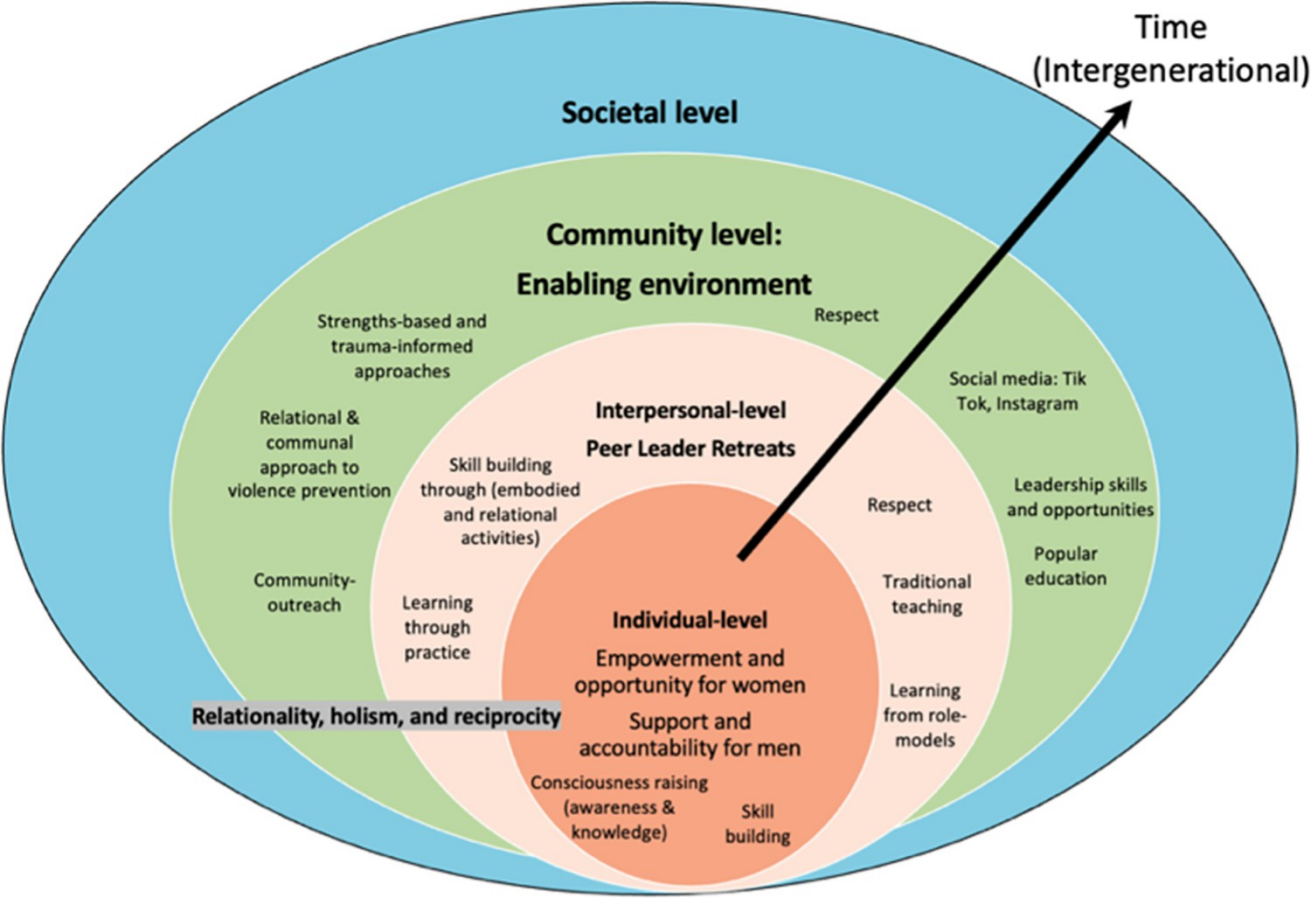
IPV prevention and healthy relationship empowerment across the ecological model amongst Indigenous and Northern youth peer leaders (adapted from Michau et al prevention of violence against women and girls model).

Qualitative findings suggest three, interconnected levels of healthy relationship promotion and violence prevention among FOXY and SMASH participants that align with social ecological approaches to IPV [26]. First, they spoke about the content and value of individual-level learning of healthy relationship knowledge and skills. Second, they spoke about specific aspects of the Peer Leader retreat as an enabling environment to learn about, and practice healthy relationship skills. Third, they spoke about the relevance of their learning for their families, communities, and across generations. In doing so, they demonstrated how they understood learning about, and enacting healthy relationships as relational, communal and intergenerational. Each of these three, interconnected layers are described below with attention to sub-themes. We adapted Michau and colleagues’ [26] socio-ecological framework for the prevention of violence against women and girls, focusing on youth empowerment at the individual, interpersonal, and community levels (see **Fig 1**).

### Individual-level: Awareness and knowledge

Three individual-level aspects of healthy relationships awareness and knowledge were identified inductively within the qualitative data. These included: (1) learning to identify healthy and unhealthy relationships; (2) new insight into being in an unhealthy relationship, together with options and skills for leaving; and (3) reflecting on one’s role in perpetrating abusive practices in dating relationships, alongside commitment to change.

### Learning about and identifying healthy and unhealthy relationships

Both FOXY and SMASH participants discussed how they learned about what constitutes a healthy and unhealthy relationship through attending a Peer Leader retreat.

**Peer Leader 1:** … we learnt… to know if it is a healthy relationship or not.

**Peer Leader 2:** We learnt how to define healthy and unhealthy relationships.

…

**Peer Leader 3:** It is easier to define what is bad, and what is healthy now.

(FOXY Peer Leader FGD, 2021)

What you guys did here educating people about what is and isn’t a healthy relationship… that helped me a lot.

(FOXY Peer Leader FGD, 2020)

I learned the signs of a healthy and unhealthy relationship and that has really helped me to have set goals for a future partner, because I definitely don’t want, I don’t think anyone wants an abusive significant other…

(FOXY Peer Leader FGD, 2020)

Commonly cited aspects of unhealthy relationships included violence ‘*I learned that hitting is not good*’ (Peer Leader FGD, FOXY 2018), alongside put downs and emotional abuse. By contrast, participants described healthy relationships as characterised by respect, communication and healthy boundaries. Participants described how learning about ‘red’, ‘yellow’ and ‘green flags’ as a helpful way to identify aspects of healthy and unhealthy relationships. The terms ‘red’, ‘yellow’ and ‘green’ flags are terms used to describe signs of dating violence and (un)healthy behaviours, with red flags being warnings, yellow flags denoting potentially concerning behaviours to monitor and green flags signs of healthy behaviour.

**Evaluator:** Do you feel like you have the skills to stay safe and healthy in your relationships and avoid violence after participating in the retreat?

…

**Participant 3:** One hundred percent…I feel like I learned more about healthy relationships … I learned what’s not healthy, say hitting and bad words and putting each other stuff and down.

**Participant 1:** What to look for in a guy and what not to look for.

**Participant 4:** Or a girl. Or a they/them.

**Participant 1:** Yes.

(FOXY Peer Leader FGD, 2020)

I think the teaching about red flags was really important because if you can spot what’s wrong in a relationship early then you are less likely to get to that point where it’s like hitting and violence and you can get out before it’s really bad. (FOXY Peer leader FGD, 2020)

**Evaluator:** Any examples of green flags?

**Participant 1:** Trust, loyalty, honesty, giving space and not being co-dependent on each other.

(SMASH Peer Leader FGD, 2020)

**Participant 1:** To respect them.

…

**Participant 2:** Ask for permission.

**Participant 3:** Communication.

**Participant 4:** Respect each other

(SMASH Peer Leader FGD, 2018)

As demonstrated by the above quotes, participants consistently described information about healthy relationships and IPV as helpful and empowering. They also spoke about how this knowledge affected them, and how it would shape their future decisions. FOXY participants in particular described how learning about signs and characteristics of an unhealthy relationship, would in turn help them to navigate relationships, or prevent getting into an unhealthy relationship altogether.

You know that it’s never okay to be hitting other people or cheating or being unloyal… I personally, from learning lots of things from FOXY about healthy relationships… I don’t think I will ever get in an (unhealthy) relationship. Or even if I do, I know that I would end it because it’s not healthy. (FOXY Apprentice FGD, 2019)

So it like taught us how to prevent getting into situations like that and how to get out of situations when we do find ourselves in them. (FOXY Peer Leader FGD, 2018)

I was always like a person to like, even if they hurt me, I would still like accept it… I know what’s wrong and what’s not wrong now. And that I need to like, help myself more. (FOXY Peer Apprentice FDG, 2021)

#### New insight into being in an unhealthy relationship and options for leaving unhealthy relationships

Learning about healthy and unhealthy relationships caused participants to reflect on the health of their current and past relationships. FOXY and SMASH participants described gaining new insights into the health of their current and previous relationships, while FOXY participants in particular spoke about gaining new insights into being in an unhealthy relationship.

When I was looking at the healthy ones (relationships), I was like *’shit—me and (name removed) have that*’. I was like ’*sick*!’.”

(SMASH Peer Leader FGD, 2018)

I learnt what a toxic relationship is (and) I realized my recent relationship was toxic. (FOXY Peer Leader FGD, 2020)

Beyond being able to identify healthy and unhealthy relationship behaviours and assess the health of their own relationships, participants described how this knowledge would help them to navigate dangerous situations and unhealthy relationships. Returning participants also discussed how they applied the knowledge and skills they learnt at FOXY in their relationships.

**Participant 1:** Yes… Yeah, I mean hopefully I don’t get into that kind of relationship, but I would know how to take care of myself and I would probably try my best to prevent it if I found that a person was like that.

**Participant 2:** …I feel like I know how to try to prevent it. Say if we have a disagreement I can go to my sisters for a night and just cool off and talk about it the next day or something like that…there are resources and ways and you (can) lead people to resources that can help with that, which is a really good thing…

(FOXY Peer Leader FGD, 2020)

**Evaluator:** Do, do you feel like you have the skills to keep safe and healthy in your relationships and avoid violence after participating in this retreat? And in what ways?

…

**Participant 1:** Like, that it’s okay to set your boundaries in a relationship, and it’s okay to say you’re not okay with something.

…

**Participant 2:** … I also didn’t know what manipulating/gaslighting was. I knew a little bit about it but not a lot about it. So after I found out what manipulating and gaslighting… I was like wow.

…

**Participant 3:** It’s like, I’m gonna like, recognize (unhealthy relationships)… I guess it’s like, it tells you how to deal with it, and how to like, get help in that situation. And more on how to recognize when you’re in one.

(FOXY Peer Leader FGD, 2021)

#### Reflecting on one’s role in perpetrating abusive practices in dating relationships, alongside a commitment to change

In addition to speaking about their experiences of victimisation, participants also reflected on their roles in perpetrating IPV. This was true for both FOXY and SMASH participants, who spoke about how learning about healthy relationships made them aware that they were behaving in unhealthy ways. This was often discussed alongside a commitment to change. Such responses were gendered: while young men spoke about learning that it was not okay to coerce or force someone sexually, both young men and women reflected on learning that it isn’t okay to perpetrate physical violence, with a focus on hitting. In some cases, girls also spoke about being aware of the risk of physical retaliation as a reason why they should not hit their partners.

No hitting your partner or abusing or demanding or forcing them to do what you want.

(SMASH Peer Leader FGD, 2018)

…I always thought like if he’s being a douche I’ll just slap him or something, but now I know that if I do that it could open a door to something really bad, so I was like maybe I won’t do that… So it like taught us how to prevent getting into situations like that and how to get out of situations when we do find ourselves in them.

(FOXY Peer Leader FGD, 2018)

### Individual-level: Skill-building

Beyond being able to identify characteristics of healthy and unhealthy relationships, and to assess the health of their own current and previous relationships, participants spoke about specific skills that they learned at FOXY and SMASH that would help them enact healthier relationships in practice. These included (1) strategies to avoid violence perpetration; (2) communication and boundary-setting; (3) increased confidence and feelings of self-worth. Participants spoke about how these skills would equip them to prevent unhealthy relationships, and navigate unhealthy situations if they emerged.

#### ‘I learned how to just breathe it out’: Identifying and navigating negative emotions

Beyond speaking abstractly about unhealthy relationship behaviours, participants spoke about strategies to avoid violence perpetration. Specifically, they spoke about learning to be more aware of their feelings, and how to calm down and express them in healthier ways. This was especially prominent with SMASH participants, who identified strategies such as breathing, taking a walk and allowing themselves to speak about their feelings:

I have switched my way of thinking because before I came to SMASH I thought what people told me, ahm like real men don’t cry, can’t show emotions, gotta be able to fight, use violence, yeah… so I always kept my emotions inside which lead me to snap at people. So now I know it’s okay to let you emotions out and real men [is] strong enough to show emotions.

(SMASH Peer Leader FGD, 2017)

**Evaluator:** Do you feel like you gained skills that will help you be able to avoid violence after participating in the retreat?

**Participant 1:** Yeah. I learned how to just breathe it out or calm my mind or put it elsewhere and be more patient.

**Participant 2:** Really grounding myself really calmed me down in the mind.…In a youth relationship they are still young and don’t know much about how to really calm down or anything.

(SMASH Peer Leader FGD, 2020)

When it was time for drum circle… it was easier to go inside yourself and recognize your feelings and not like, just if you get mad at someone then your first reaction is to hit someone because you can notice what you’re feeling like instead of just wanting to hit.

(FOXY Peer Leader FGD, 2018)

#### Building confidence and self-worth

Participants also spoke about how retreat participation built their confidence and sense of self-worth. FOXY participants in particular reflected on how valuing themselves more made them more resilient and better able to avoid, navigate and leave unhealthy relationships. Further, they described gaining better communication and boundary-setting skills as important in both preventing, and responding to violence in relationships:

Now I can say I’m worth more than being in an abusive relationship.

(FOXY Apprentice FGD, 2019)

**Participant 1:** I guess I’ve changed a lot [laughs]. I think I value myself more… I find myself more important, and definitely more confident. And more open with others…

**Participant 2:** Same with like (name removed) said, like I’m more confident and open.

(FOXY Peer Apprentice FGD, 2020)

I learned a lot. Like if he calls me something, then I got boundaries and I can tell him not to call me that.

(FOXY Peer Leader FGD, 2018)

**Participant 1:** Have really good communication. Like talk about the things that are bothering you in a calm way. Just sit down and talk to each other for maybe an hour at least once a week.

**Participant 2:** Stand up for yourself if they keep using verbal abuse or something like that. (FOXY Peer Leader FGD, 2020)

### Interpersonal-level: Learning by seeing and doing in Peer Leader retreat environment

Participants also spoke about aspects of the retreat environment that were instrumental in learning about healthy relationships and developing healthy relationships skills, including communication and healthy boundaries. The Peer Leader retreat model was discussed as central to interpersonal learning about healthy relationships in three ways. First, participants learned from how camp leaders and facilitators–some of whom attended the retreats with their families–modelled healthy relationships with each other. Second, they learned through activities such as acting, charades and resource-mapping which provided embodied and experiential knowledge about enacting healthy relationships and responding to unhealthy practices. Third, they engaged with the retreat environment as a space to practice identifying and enacting healthy relationship skills platonically with one another.

#### Learning from retreat leadership role modelling

Peer leaders (participants), peer apprentices and facilitators alike spoke about how seeing healthy relationships enacted and role modelled by staff members was an important source of learning at Peer Leader Retreats:

Having both of you guys (facilitator couple) there role modelled a very healthy relationship and that was a great example to have around and all of the kids mentioned that. They always had something to say about that. (SMASH Facilitator FGD, 2018)

Like there are so many moments when the kids were looking at them (facilitators) and being like *’oh man that’s going to be me some day—that’s gonna be me*.*’* Cause they were teaching together, they were learning together, they were having fun together. They respect each other.

(FOXY Facilitator FGD, 2018)

#### Learning from retreat activities

Participants spoke about how specific activities, namely healthy and unhealthy relationship charades, provided them with embodied experiences of, and practice with responding to unhealthy relationship experiences and enacting healthier practices:

**Participant 1:** I really liked the charades and stuff. I thought that was really educational.

**Participant 2:** The unhealthy, healthy FOXY activity was a good way to, even without hearing, to pinpoint what type of relationship you are dealing with, which would make it even easier to identify yourself, so I feel that I would be able to identify an unhealthy relationship sooner.

…

**Participant 3:** Yeah, I learned that it isn’t ok for your partner to hit you if you’ve said something in front of his or her friends.

…

**Participant 2:** I actually learned that many of my relationships were not at all very healthy and I didn’t know that a lot of these things were traits of unhealthy relationships, so I’m going to try and be a lot more careful when it comes to choosing friendships or relationships. (FOXY Peer Leader FGD, 2020)

You could act out relationships, good relationships. (FOXY Peer Leader FGD, 2019)

FOXY participants also discussed the importance of activities where they mapped social and communal resources that helped them to identify different sites of social support in their own lives, and to understand how social support can help when navigating, and leaving unhealthy relationships. These supports included family, friends, programs and community resources. Participants also discussed the connections they made with one another, and FOXY as sites of social support.

There can be arguments and more pressure into sexual relationships… there are resources and ways, and you lead people to resources that can help with that, which is a really good thing. Some people are scared to go to people if they’re in those kinds of relationships and you showed us there’s no reason we have to be scared. We can prevent it ourselves.

(FOXY Peer Leader FGD, 2020)

I think it was really great to share stories… because at home I don’t have that. I have a support system, but I don’t have where I can freely talk… I’ve really liked it cause I felt safe while doing it and I felt like I wasn’t judged and I felt listened to… so that was nice to come out here and do that and I feel like I am ready to face another challenge at home.

(FOXY Peer Leader FGD, 2020)

**Participant 1:** Always text your friend if you ever try to break up with someone… Or always have your friends to come check on you.

**Participant 2:** Yeah like let them know if you’re going to do something just so they like know so they can kind of like have your back if anything happens.

(FOXY Peer Leader FGD, 2018)

#### Learning leadership through practice in the retreat environment

The Peer Leader model situated participants within a leadership role, creating a sense of responsibility and empowerment. Participants described the retreats as an opportunity to role model healthy relationship practices, and to support others to build healthy relationship skills. This was especially prominent for Peer Apprentices, who were returning to the camp in leadership roles:

**Peer Apprentice 1:** Everybody knows now (about healthy relationships). At the start of the week nobody knew anything.

**Peer Apprentice 2:** I feel like people definitely learned the stuff, because I overheard people saying “*oh wait*, *that’s not a healthy thing*”.

**Peer Apprentice 3:** …I noticed a lot of people say “*hey*, *I don’t like when you do this*”, and they would talk to them about their boundaries. Like “*hey*, *stop that right now*.”

(FOXY Peer Apprentice FGD, 2021)

… I helped a couple of them express their anger toward other people through either words or just ignoring it all together instead of throwing hand (at the) first opportunity. (SMASH Peer Apprentice FGD, 2021)

If you notice your friend is angry take them to the side and check in with them. Check up on one another occasionally.

(SMASH Peer Leader FGD, 2020)

Notably, participants extended their experiences of enacting healthy practices at retreats to a commitment to role modelling healthy relationships when they returned home. Participants frequently spoke about how they wished to empower others in their communities to also have healthy relationships through sharing what they had learned.

### Community level: Empowering healthy relationships communally, relationally and intergenerationally

Beyond the direct personal benefits of the healthy relationship knowledge and skills learned at Peer Leader retreats, participants described how they view enacting and promoting healthy relationships as relational, reciprocal and in service to their communities across generations. Three sub-themes were present: (1) empowering healthy relationships communally through using their new knowledge and skills to support others; (2) Strengths-based, trauma-informed, culturally-grounded approaches to empowering healthy relationships alongside contextualising violence within a history of colonial violence, dispossession of land and culture, and residential schools; and (3) responsibility, respect and intergenerational healing: enacting healthy relationships as a form of respect to Elders, families and peers, and generations still to come. Each sub-theme is presented and described below, and is accompanied with illustrative quotes.

#### Empowering healthy relationships communally

Participants discussed how their commitment to healthy relationships extended to a sense of communal responsibility. FOXY participants in particular demonstrated a desire to improve the well-being of their communities by sharing their new relationships knowledge and skills with friends, families and community members when they returned home. They described planning community projects to share their knowledge and skills in-person, as well as over social media platforms like Instagram and TikTok.

I feel great about my community project. It’s about unhealthy relationships and I really think people need to know more about it… there is a lot of abuse going through relationships… and a lot of negativity between certain people, so it is good to go out and pursue healthy relationships because that’s what needs to be done… I can help somehow with it…

(FOXY Peer Leader FGD, 2020)

The relationship things, like unhealthy relationship and healthy relationships. I learned a lot with that… I want to bring it back to my community… because my community, people have a lot of unhealthy relationships.

(FOXY Peer Leader FGD, 2021)

**Participant:** I feel like I could spread awareness for toxic and healthy relationships. And how I could teach people about the signs, and how they could get out of it…

(FOXY Peer Leader FGD, 2021)

**Evaluator:** How do you feel about doing your community projects, and do you think it will have an impact on your community?

…

**Peer Leader 1**: I think it (community project) will help a lot of woman in need at the woman’s shelter…I am feeling confident about it.

**Peer Leader 2:**., It will be a video posted on Instagram… I am confident it will go good.

**Peer Leader 3:** I am hoping I will be able to create an Instagram to promote sexual health and healthy relationships…

(FOXY Peer Leader FGD, 2021)

#### Strengths-based, trauma-informed, culturally-grounded approaches to empowering healthy relationships

Participants affirmed the use of a strengths-based, trauma-informed approach to IPV prevention and healthy relationship promotion. In particular, they highlighted the complex relationships between violence perpetration ideation, shame and trauma within the context of the historic and ongoing violence of colonisation and residential schools. They suggested that a strengths-based focus on providing healthy relationship knowledge and skills was empowering and affirmed the value of Indigenous frameworks such as medicine-wheel teachings and art-based approaches:

I really think it is important to destigmatize what we call “*toxic*” and “*toxicity*” in relationships. We are living past residential schools, so there were some unhealthy ways picked up. We have to destigmatize it. Once you think “*I am bad*, *I’m not as good as other people*”… So many of our participants do experience or witness domestic violence, so that can be really hard and confusing. “*I guess we are all just bad now*”, just really hitting home that if you’ve had ways that are not healthy to get your needs met in the past, doesn’t make you a bad person.

(FOXY Facilitator FGD, 2021)

**Participant 1:** I think also teaching healthy coping mechanisms, because sometimes violence is a coping mechanism, it’s not a very healthy one, but it is one and teaching healthy alternatives to avoid that, because I do writing now instead of getting in fights with people at school.

**Participant 2:** So yeah learning what works for you. Like we did that medicine wheel and we wrote and that was really important to know…

(FOXY Peer Leader FGD, 2020)

#### Responsibility, respect and intergenerational healing

FOXY and SMASH participants, and particularly young men spoke about the importance of respect in their learning about healthy relationships. Respect was discussed as part of a commitment to enacting healthy relationships, and also more broadly as a commitment to change.

Like respect people, your partner… The SMASH Retreat is really helpful for young people so they don’t grow up to be mean to everyone.

(SMASH Peer Leader FGD, 2018)

Alongside this, participants described being respectful and forming healthy relationships as a commitment to their families, Elders, communities and future generations. Within these narratives they demonstrated that they understand wellbeing and healthy relationships as relational, communal and intergenerational.

I feel like I’ve changed in the way of just like having more respect for more people and having more knowledge… I do take a lot of the rape jokes and shit seriously. It’s about people I know, but I do feel like I’ve changed and when I go home I just kind of want to act more respectful to most of my Elders back home.

(SMASH Peer Leader FGD, 2018).

Before I came here I had a lot of anger issues. I would throw attitude with my mom and punches at my dad, but now that I’m here I feel more relaxed and I feel more accepted and I feel like I have more respect to give.

(SMASH Peer Leader FGD, 2020)

I really, really like what (Elder name) said. She said your future children are already inside of you so don’t do anything that you wouldn’t want them to see or do.

(FOXY Peer Leader FGD, 2019)

## Quantitative results: Participant characteristics

**Table 2** displays the sociodemographic characteristics of participants enrolled in the Peer Leader Retreats. Our analysis included 240 participants who had ever been in an intimate relationship (87 young men and 144 young women) about their attitudes towards IPV. The mean age of the respondents was 14.5 years (SD = 1.3), and most participants (64.2%) were from the FOXY retreat. Nearly seventy percent of participants were Indigenous. Most participants identified as heterosexual (66.4%), while sexually diverse participants accounted for 33.6% of the study sample.

**Table 2:** Sociodemographic characteristics of quantitative participants (n = 240).

Variable	Frequency n (%)
Gender	
Male	87 (36.2%)
Female	144 (60%)
Other	9 (3.8%)
Retreat	
FOXY	154 (64.2%)
SMASH	86 (35.8%)
Age (mean/SD) ^n = 2^	14.5 (1.3)
Sexual orientation	
Lesbian	5 (2.1%)
Gay	2 (0.8%)
Bisexual	45 (18.9%)
Straight	158 (66.4%)
Queer	5 (2.1%)
Other	23 (9.7%)
Ethnicity	
Aboriginal Indigenous	167 (69.6%)
White/Caucasian	57 (23.8%)
Others	16 (6.6%)

### Quantitative results: IPV attitudes

Cronbach’s alpha was computed separately by gender, and the scale had an acceptable internal consistency with Cronbach alpha of 0.73 (male: 0.69; female: 0.74) for the 10 items’ scale, and 0.90 (male: 0.90; female: 0.86) on the 11-item scale.

As shown in Table 3, respondents from 2018 to 2020 indicated low attitudes accepting of IPV. The mean level of attitudes accepting of IPV for the entire sample was 1.41, and most study participants (88%) stated higher acceptance of violence as either “completely wrong,” or “mostly wrong”. IPV attitude scores among young men were not significantly different between pre-retreat (mean = 1.39, SD = 0.65) and post- retreat (mean = 1.49, SD = 0.76). However, among FOXY participants, approval of attitudes toward accepting IPV reduced significantly after the retreat (mean pre-retreat = 1.41, SD = 0.62; mean post-retreat = 1.28, SD = 0.46; p = 0.0019).

**Table 3:** Mean levels of endorsement of attitudes toward IPV before and after training by gender, 2018–2020 (N = 173) *Pvalues were generated using paired T-tests comparing pre- and post-test scores*.

Items	Mean of approval (1 to 6)						
	Total (173)		Male (62)			Female (107)	
	Before	after	Before		After	Before	After
It is OK for a person to hit their boyfriend/ girlfriend/ partner if the partner did something to make them mad	1.3	1.28		1.37	1.47	1.27	1.18
It is OK for a boy to force a girl to have sex if she got him sexually excited	1.27	1.31	1.13		1.41	1.30	1.23
Hitting a boyfriend/girlfriend/partner may be OK	1.19	1.22	1.16		1.26	1.21	1.2
It is OK for a person to hit their boyfriend/ Girlfriend/partner if they insulted them in front of friends	1.35	1.28	1.40		1.44	1.31	1.19
A person sometimes deserves to be hit by the person they date	1.39	1.36	1.39		1.67	1.41	1.19**
Sometimes a person has to hit their boyfriend/ girlfriend/partner to get them back under control	1.66	1.60	1.73		1.84	1.64	1.47*
It is OK for a person to hit their boyfriend /girlfriend /partner if they partner hit them first	1.75	1.69	1.52		1.58	1.91	1.73
It is OK for a boy to force a girl to have sex if he paid for all the costs of a date	1.33	1.14*	1.43		1.31	1.25	1.05*
A person who makes their boyfriend/girlfriend/ partner jealous on purpose, deserves to be hit	1.42	1.35	1.4		1.5	1.43	1.26
**Attitude toward IPV (average)**	1.41	1.36	**1.39**		**1.49**	**1.41**	1.28**

1 = Completely wrong 2 = Most times wrong 3 = More wrong than true.

4 = More true than wrong 5 = Most times true 6 = Completely true.

*- P value <0.05

**-P value < 0.001.

For study participants enrolled from 2018 to 2020, pre- and post-training changes in boys’ and girls’ on approval levels for each item in the scale were similar, except for three items among girls (Table 3). After the retreat, FOXY participants showed significantly lower acceptance attitudes for the following three scale items: “A person sometimes deserves to be hit by the person they date” (1.41 vs 1.19, P< 0.001), “Sometimes a person has to hit their boyfriend/girlfriend/partner to get them back under control” (1.64 vs 1.47, P< 0.05), and “It is OK for a boy to force a girl to have sex if he paid for all the costs of a date (1.25 vs 1.05, P< 0.05).

Likewise, for the 2021 participants, FOXY participants scored significantly lower for their overall attitude toward acceptance of IPV after retreat, pre- (mean = 13.21, SD = 3.64) and post-retreat (mean = 12.29, SD = 2.63) (p = 0.0256). As shown in Table 4, SMASH participants’ attitudes toward accepting IPV also reduced, though was not significantly different between pre- (mean = 16.38, SD = 6.18) and post-retreat (mean = 14.67, SD = 4.49) (p = 0.1592).

**Table 4:** Mean levels of endorsement of attitudes toward IPV before and after training by gender, 2021 (N = 63).

Items	Mean of approval (1 to 6)					
	Total (63)		Male (24)		Female (34)	
	Before	after	Before	After	Before	After
A boy angry enough to hit his dating partner must love them very much.	1.21	1.12	1.5	1.13	1.03	1.11
Violence between dating partners can improve the relationship	1.24	1.21	1.5	1.38	1.11	1.14
Girls sometimes deserve to be hit by persons they date	1.11	1.06	1.29	1.13	1.00	1.00
A girl who makes her dating partner jealous on purpose deserves to be hit	1.27	1.17	1.38	1.25	1.24	1.11*
Boys sometimes deserve to be hit by their dating partner	1.33	1.19	1.58	1.5	1.22	1.03
A girl angry enough to hit her dating partner must love them very much	1.14	1.12	1.25	1.17	1.08	1.08
There are times when violence between dating partners is okay	1.39	1.29*	1.67	1.54	1.27	1.16
A boy who makes his dating partner jealous on purpose deserves to be hit	1.41	1.29	1.63	1.5	1.32	1.16*
Sometimes violence is the only way to express your feelings	1.38	1.19*	1.5	1.33	1.35	1.11*
Some couples must use violence to solve their problems	1.30	1.14**	1.58	1.29*	1.16	1.05*
Violence between dating partners is a personal matter and people should not interfere	1.37	1.34	1.5	1.46	1.33	1.28
**Attitude toward IPV (sum)**	14.18	13.15*	**16.38**	**14.67**	**13.21**	12.29*

1 = Completely wrong 2 = Most times wrong 3 = More wrong than true.

4 = More true than wrong 5 = Most times true 6 = Completely true.

*- P value <0.05

**-P value < 0.001.

## Discussion

We explored the role of Peer Leader Retreats in supporting healthy relationship knowledge, skills and efficacy amongst Indigenous and Northern young people in the NWT. Findings demonstrate the potential role of Peer Leader retreats in improving IPV and healthy relationships knowledge and skills, alongside effective aspects of programming. Findings also point to the communal, relational and intergenerational meaning of such knowledge for young men and women. Results build on previous findings from this same program, which demonstrated empowerment-related outcomes [23], improved sexual health knowledge, safer sex self-efficacy, and increased resilience [24] to show that peer and land-based approaches hold promise in IPV prevention.

The perspectives and experiences of FOXY and SMASH participants–gathered from focus groups and triangulated with surveys–point to important areas for strengths-based, healthy relationships programming with Indigenous and Northern young people. First, findings affirm the value of strengths-based peer leader approaches that focus on teaching about healthy relationships, providing skills and creating opportunities to see, enact and share about healthy relationships. Such an approach stands in contrast to negative, deficit, and risk-focused youth violence discourses [16]. Further, findings affirm the value of a trauma-informed approach, which acknowledges intergenerational trauma, including from violence of colonisation and residential schools. In doing so, findings respond to the paucity of literature on strengths-based and culturally-informed approaches to intimate violence prevention among Indigenous and Northern young people, which may have relevance for both primary, and secondary IPV prevention. They contribute to a growing call for the importance of culturally, socially and historically responsive violence prevention and healing strategies for youth in Canada [4]. They also bolster a growing evidence on work with Indigenous and northern young people in the Northwest Territories on sexual health, relationships, health and wellbeing [36–38].

While qualitative findings demonstrate the value of violence prevention and healthy relationship programming with Indigenous and Northern young people, results were gendered and suggest that programmatic aspects may affect young men and women differently. While this study was not designed to test effectiveness of the Peer Leader Retreats in impacting outcomes, we did note among young women significant reductions in accepting attitudes toward IPV following retreat attendance. Qualitatively, young women were strongly committed to sharing what they had learnt with other women, demonstrated uptake of leadership roles and a communal commitment to violence prevention. Young men discussed the importance of being able to identify and express emotions as an important aspect in preventing violence perpetration, and resonated strongly with learning about values of respect. The gendered nature of these findings point to a need to further explore gender-tailored healthy relationship and IPV prevention programming, including the possibilities and processes through which strengths-based and trauma-informed Peer Leader Programming may interrupt and re-signify hegemonic masculine norms [39]. This is especially pertinent given a relatively limited research and programmatic focus on boys and men [40,41]. Such an exploration would be well-placed and timely, given the damaging and far-reaching impacts of western patriarchal colonial masculine norms which have disrupted more egalitarian gender norms within Indigenous and Northern contexts [42].

Findings also demonstrate the value of peer-models in healthy relationship programming. In a previous study, we found that role modelling healthy behaviours and relationships was considered to be a form of leadership through action [23]. Similarly, a school-based, culturally-relevant peer programme for Indigenous youth in Ontario documented improved communication, strengthened relationships, and connectedness with peers [43]. Connectedness–including belonging, caring and support [44]–with peers, family and community is protective against violence [45]. Findings from this paper contribute to this evidence in the North, and provide insight into how young Indigenous and Northern women take on leadership roles in IPV prevention and the promotion of healthy relationships. In particular, FOXY participants discussed improving healthy relationship knowledge as something highly relevant to their communities, and described feeling excited and empowered to share what they learnt with others through community projects. This finding suggests that a motivator for enacting and promoting healthy relationships is feeling empowered through knowledge and a leadership role to enact positive change. Peer leaders interpreted violence prevention and healthy relationship promotion as a relational responsibility, extending beyond their own intimate relationships to the health of their communities. In this way, they demonstrated that they viewed their commitment to healthy relationships as communal, relational and intergenerational.

Findings demonstrate the broad applicability of Michau and colleagues’ [26] socio-ecological framework for the prevention of violence against women and girls, mapping aspects across inter-related ecological systems in creating relationships free of violence. Findings from this study suggest that Peer Leader Retreats supported violence prevention at individual, interpersonal and communal levels. At the individual level are empowerment and opportunities for women, and support and accountability for men, developed through awareness and knowledge (consciousness-raising) as well as skills for healthy relationships and IPV prevention. The interpersonal level includes the Peer Leader Retreat environment, inclusive of learning from role-models, skill-building through embodied and relational activities, traditional teachings and learning through practice. At the community level, we note enabling environments inclusive of leadership skills and opportunities, relational and communal violence prevention approaches, community-level outreach including social media (e.g., TikTok, Instagram) and popular education. We also include strengths-based and trauma-informed approaches as part of an enabling environment. Given the nature of data collected, we did not engage enabling aspects of the broader societal level of the model. While this framework was broadly applicable, there were notable distinctions in study findings which are reflected in the adapted model.

First, participants engaged these levels relationally, and as a continuum from individual consciousness-raising and skill building outwards towards the creation of enabling environments and collective empowerment for social change. Second, they demonstrated that they understood promoting healthy relationships and preventing IPV as a communal, relational and intergenerational commitment, encompassing care and respect for Elders and future generations. For this reason, we have added an arrow across spheres to demonstrate the intergenerational and temporal nature of empowering heathy relationship learning and skills-development. We note the presence of relationality, holism and reciprocity as core enablers across domains. Last, findings demonstrate the value of strengths-based, culturally-grounded and trauma-informed programming. Participants engaged respect–within the ‘interpersonal’ category of the Michau model–as well as holistic, relational and communal notions of well-being as motivators towards healthy relationships. We amend the model to include approaches that value reciprocity and holism as enablers across individual, interpersonal and community levels. This finding builds upon literature that documents how building culturally relevant approaches of holism, reciprocity and relationality into research and practice with Indigenous and Northern young people is supportive of health and well-being [46–48].

There are several study limitations. There was no control group, so it is not possible to determine causality and attribute changes in outcomes to intervention participation. As the sample size per retreat was small, we pooled data across several retreats, and this could obscure differences that may have happened between retreats. We did not power the study to evaluate changes in outcome variables; the study design reflected a community-based approach to building skills among youth and the ideal number of youth were determined by the FOXY and SMASH programmes. IPV attitudes were self-reported and therefore subject to social desirability bias, which we limited by pilot-testing and reviewing the surveys. Further, IPV attitudes were measured using two different scales, which impacted comparability across retreats. Selection bias may be present in our sample, whereby youth who were more attuned to issues of violence prevention and already identified as leaders might be more likely to participate in retreats. Last, our convenience sample potentially overrepresented female participants.

Despite these limitations, this study provides a unique contribution to the small but crucial literature on strengths-based, empowering IPV prevention and healthy relationship programming with Indigenous and Northern young people, shining valuable light on an existing, strengths-based and culturally-informed model for Indigenous and Northern youth in Canada’s Arctic. Findings thus have relevance for policy and programming, amplifying the call for culturally-grounded, locally- informed and community-oriented IPV prevention within an important, yet underserved group.

## Conclusions

This multi-methods study documented how Peer Leader programming can empower Indigenous and Northern young people, their families and communities with healthy relationship knowledge and skills. Findings suggest that the effects of such programming are gendered, and that components of effective violence prevention programming with adolescent boys and young men merit further exploration.

Findings demonstrate the possibilities of peer models to encourage healthy relationships, suggesting that embodied learning and leadership opportunities are particularly effective in equipping youth with violence prevention and healthy relationship skills. Young women in particular demonstrated a commitment to sharing knowledge and skills about healthy relationships in their communities, including through novel approaches such as social media. For young men, teachings of respect, alongside support to feel and express feelings was described as empowering and transformative. Participants regardless of gender, demonstrated their belief that healthy intimate partner relationships also have communal and relational functions, and are an investment for future generations.

Findings contribute to the emergent evidence base on the value of strengths-based, culturally-responsive violence prevention programming, and suggest that such programming is well-placed within peer-led, community initiatives.

## Supporting information

S1 Checklist (DOCX)

S1 File (DOCX)
